# Psychological adjustment to infertility and its effect on mental health in women

**DOI:** 10.1192/j.eurpsy.2025.575

**Published:** 2025-08-26

**Authors:** M. Mitrović, N. Ćirović, J. Opsenica Kostić, I. Janković, M. Guberinić, M. Spasić Šnele, M. Trenkić

**Affiliations:** 1 Department of Psychology, Faculty of Philosophy, University of Niš; 2 Faculty of Medicine, University of Niš, Niš, Serbia

## Abstract

**Introduction:**

Infertility represents a serious biopsychosocial crisis. Faced with infertility in a society that emphasizes parenthood as a desirable role, and driven by their own desire to become a parent, a person is forced to (re)define themselves as an adult and find their place in society. Research shows that women are more affected by this issue. Firstly, medical treatment for infertility is more uncomfortable for women than for men. Moreover, in most societies, women are considered the responsible partner for reproduction. The experience with this problem can be traumatic enough to become a reference point for identity and for attributing meaning to other experiences in one&rsquo;s life.

**Objectives:**

This research aimed to examine the mediating role of psychological problems caused by infertility in the relationship between self-stigma and the degree of integrating trauma (i.e. infertility) into one&rsquo;s identity, on the one hand, and the level of depression, on the other.

**Methods:**

The study involved 197 women with difficulties conceiving who had not yet become mothers, aged between 27 and 47 years (M=37.7; SD=5.1). The following questionnaires were used: the Female Infertility Stigma Instrument (ISI-F), designed to assess self-stigma in women facing infertility. The Centrality of Event Scale (CES) measures integrating trauma into one’s identity (adapted here to measure the integration of infertility into one’s identity). The Psychological Evaluation Test for Infertile Couples (PET), is aimed at assessing psychological problems in various aspects of life caused by infertility, and the Patient Health Questionnaire (PHQ-9), is used to assess the degree of depression.

**Results:**

The results show that the zero-order correlations between the predictors, mediators, and criterion are positive and significant. Mediation analysis shows that problems caused by infertility mediate the relationship between self-stigma and the level of depression (indirect effect: ß=.292, p< .001, 95%CI = .199 - .411), and between the integration of trauma (i.e., infertility) into one’s identity and the level of depression (indirect effect: ß=.147, p<.001, 95%CI = .083 - .226). In both cases, full mediation is observed. Tables and figures will be added to the poster if accepted.

**Conclusions:**

The results indicate that the degree of internalized stigma and the integration of infertility into one’s identity are significant for a person’s adjustment to this problem and that, together with the level of psychological problems caused by infertility, have an effect on mental health. The study underscores the “traumatic” potential of infertility and the effect of this issue on identity and mental health. The findings could also serve as guidelines for designing psychological interventions, which should focus on the problem of self-labeling, self-blame, and the redefinition of oneself as an adult.

**Disclosure of Interest:**

M. Mitrović Grant / Research support from: This research was supported by the Science Fund of the Republic of Serbia, #GRANT No 1568, Identity Crisis in Women Facing Infertility: Mixed Methods Approach – InsideMe, N. Ćirović Grant / Research support from: This research was supported by the Science Fund of the Republic of Serbia, #GRANT No 1568, Identity Crisis in Women Facing Infertility: Mixed Methods Approach – InsideMe, J. Opsenica Kostić Grant / Research support from: This research was supported by the Science Fund of the Republic of Serbia, #GRANT No 1568, Identity Crisis in Women Facing Infertility: Mixed Methods Approach – InsideMe, I. Janković Grant / Research support from: This research was supported by the Science Fund of the Republic of Serbia, #GRANT No 1568, Identity Crisis in Women Facing Infertility: Mixed Methods Approach – InsideMe, M. Guberinić Grant / Research support from: This research was supported by the Science Fund of the Republic of Serbia, #GRANT No 1568, Identity Crisis in Women Facing Infertility: Mixed Methods Approach – InsideMe, M. Spasić Šnele Grant / Research support from: This research was supported by the Science Fund of the Republic of Serbia, #GRANT No 1568, Identity Crisis in Women Facing Infertility: Mixed Methods Approach – InsideMe, M. Trenkić Grant / Research support from: This research was supported by the Science Fund of the Republic of Serbia, #GRANT No 1568, Identity Crisis in Women Facing Infertility: Mixed Methods Approach – InsideMe.

